# Global Aging & Geriatric Experiments in Bipolar Disorder (GAGE-BD): Building a Global Consortium Benefiting People with Bipolar Disorder in Later Life

**DOI:** 10.1007/s11920-025-01593-w

**Published:** 2025-03-11

**Authors:** Melis Orhan, Laura Montejo, Martha Sajatovic, Lisa Eyler, Annemiek Dols

**Affiliations:** 1 Institute of Clinical Psychology, Leiden University, Leiden, The Netherlands; 2 Bipolar and Depressive Disorders Unit, Hospital Clinic de Barcelona, Institut d’Investigacions Biomèdiques August Pi i Sunyer (IDIBAPS), Institute of Neurosciences (UBNeuro), Centro de Investigación Biomédica en Red de Salud Mental (CIBERSAM), Instituto de Salud Carlos III, Madrid, Spain; 3 University Hospitals Cleveland Medical Center, Case Western Reserve University School of Medicine, Cleveland, Ohio USA; 4 Department of Psychiatry, University of California San Diego, La Jolla, California USA; 5 Desert Pacific Mental Illness Research Education and Clinical Center, Veterans Affairs San Diego Healthcare System, San Diego, California USA; 6 Department of Psychiatry, UMC Utrecht Brain Center, University Medical Center Utrecht, Utrecht, The Netherlands

**Keywords:** Bipolar disorder, Older adults, Older age, Global dataset, Aging

## Abstract

**Purpose of Review:**

Findings from the Global Aging & Geriatric Experiments in Bipolar Disorder (GAGE-BD) project, including sociodemographic and clinical information from older age bipolar disorder (OABD) and healthy participants around the globe (approximately *N* = 5000) were reviewed. Data was collected in multiple waves to create a large integrated dataset.

**Recent Findings:**

BD does not seem to fade with age. BD subtype and early/late onset did not show significant differences in daily functioning. Physical comorbidities were more frequent in OABD compared with controls. Women with OABD had an earlier age at onset and more psychiatric hospitalizations.

**Summary:**

GAGE-BD is the largest OABD cohort. Dataset results offer a unique and comprehensive resource for understanding the long-term trajectory of BD and the specific needs of this population. Findings are vital for guiding future research and improving care strategies for aging individuals with BD.

**Supplementary Information:**

The online version contains supplementary material available at 10.1007/s11920-025-01593-w.

## Introduction

Population aging is increasing, with the proportion of individuals aged 60 years or over growing in tandem with declines in the proportion of individuals under the age of 15, particularly in Western and East Asian nations [1]. In alignment with this demographic shift, approximately one-quarter of individuals with bipolar disorder (BD) are aged 60 and over [2]. Consequently, clinicians and healthcare systems must be equipped to provide care and services to individuals with BD throughout their lifespan, with particular attention to the specific needs of an expanding older population.

Older age bipolar disorder (OABD) pertains to individuals diagnosed with BD who are aged 50 years and over. The International Society for Bipolar Disorders (ISBD) Task Force on OABD has established this age threshold [3], arguing that BD constitutes a severe mental illness associated with a reduced life expectancy of approximately 10–20 years [4, 5]. Furthermore, in BD, biological age is often advanced relative to chronological age, contributing to premature aging [6].

OABD represents a distinct and complex more intricate subgroup of BD, characterized by prevalent cognitive deficits [7, 8], an elevated risk of dementia [9], impaired psychosocial functioning [10], frequent physical comorbidities [11], and premature mortality [4, 5, 12]. Several aging-related factors further exacerbate outcomes in OABD, including shrinking social networks, diminished support from friends and family, cumulative down-steam effects of lifestyle choices, reduced mobility, declining physical health, and other age-related issues. Although OABD does not resolve or “burn out,” epidemiological studies suggest that BD affects 0.5–1.0% of older adults, a prevalence lower than the 1.4% observed in individuals aged 18 to 44 years [13]. The absolute number of OABD patients is projected to increase due to population aging.

A literature review and expert consensus report [3], along with a survey of guideline recommendations for OABD conducted by the ISBD Task Force on OABD [14], revealed a significant lack of evidence-based and specific guidelines specific to OABD. To address this gap, the Global Aging & Geriatric Experiments in Bipolar Disorder (GAGE-BD) project was initiated in 2018 [15]. Funded initially by the Bowden Massey Strategic Research Initiative in Bipolar Disorder Award, and later supported with philanthropic and industry initiatives, the project now includes over 20 international investigators from multiple study sites across the Americas, Africa, Asia, Europe and Oceania and two experts by experience. The GAGE-BD dataset contains sociodemographic and clinical information from OABD outpatients, elders with other mood and with psychotic disorders as well as healthy participants. The first two waves of the GAGE-BD project consisted of approximately 2600 individuals with BD as well as 1600 healthy controls [16].

In current review, we will mainly focus on findings from the GAGE-BD cohort and where necessary, these will be supplemented with findings from other research groups that have focused on OABD.

## Methods of the GAGE-BD Project

To address the gap in knowledge about aging with BD and to create a powerful integrated dataset, GAGE-BD began soliciting data contributions in 2019 with the following requirements: at least a quarter of participants over age 50, includes mood symptom severity ratings and demographics, and all identifiers removed (see gage-bd.org for more information). All available coded and numeric data were solicited, including from comparison participants without mental health conditions; data were contributed after securing necessary data use agreements and human subject approvals. Up until now, we have collected findings distilled from the first two waves. The first wave (W1) included 19 studies from 17 sites; the second wave (W2) included 19 studies from 16 sites (including additional data from some wave 1 studies).

The GAGE-BD team is currently in the process of acquiring a third “wave” of data (W3) that will engage in the order of 8–10 new sites. The types of samples/studies were varied and included both cross-sectional and longitudinal designs (all analyses to date have utilized data from only the baseline assessments of longitudinal studies); observational and interventional studies; clinical research studies, clinical case series, and epidemiological studies (see Supplemental Table 1). Because contributed data were archival or from ongoing studies, the inclusion and exclusion criteria for studies varied (see Supplemental Table 2). In addition, across the studies, data were collected using different instruments and coded differently for each study. Thus, harmonization and cross-walking of data was necessary prior to inclusion in the integrated dataset. For example, using previous cut-points from the literature, we created depression severity categories with definitions unique to each of four instruments used across studies and re-coded the continuous data into severity bins [17].

The total sample size of the GAGE-BD dataset includes currently approximately 5,000 cases, around 3,500 OABD patients and 1,400 within the non-BD group including both healthy controls and cases with other mental health diagnoses. There are 211 variables in the integrated dataset with varying numbers of participants for each variable. Covered data domains include: study meta-data, demographics, clinical characteristics, course of BD illness– symptoms, course of BD illness– episodes, course of non-BD psychiatric illness, family history, current illness severity, current pharmacological treatment, physical health, and cognitive performance (see https://cwru-non-academic.github.io/varicat/visualizations/integrated_gage.html for more details). The BD sample has an average age of 54.3 (SD = 14.5) and age ranges from 17 to 95 with 69% over the age of 50. Women make up 60% of the BD sample; 75% have BD type I. Depression and mania symptoms are absent or mild in most participants.

## Results from the GAGE-BD Project

### Clinical Presentation

One of the major efforts of GAGE-BD is to comprehensively characterize the evolution of clinical symptom patterns and mood trajectory with advancing age. Data regarding the differences in patterns of clinical manifestations between younger and older patients are mixed and inconclusive. Initial GAGE-BD findings from W1, suggested that both BD manic and depressive symptom severity appear to be reduced in older age [18]. Also, in the replication study in W2 it was found that older age was significantly associated with slightly less severe symptoms of mania while depressive symptoms were not found to be related to older age [19].

Even within OABD, approaches have been made to understand the effect of different age ranges on clinical manifestations. Thus, the GAGE-BD sample has been studied by dividing it into three different age groups: the youngest group (< 50), the middle age group (50–69), and the oldest group (> 70), and it was observed that the oldest group had the lowest levels of manic and depressive symptoms [20]. The overall psychiatric burden did not differ between the oldest and middle groups, but the youngest group showed the greatest overall psychiatric burden. These findings indicate that the relationship between symptom severity and age might be non-linear. In another GAGE-BD study, it was found that varying severity in BD symptoms was relatively common (70%). The American Psychiatric Association’s Diagnostic and Statistical Manual (DSM-5) includes a ‘with mixed features’ specifier that applies to a manic or hypomanic episode with three or more depressive symptoms (or to a depressive episode with three or more manic symptoms) [21]. It was found and that OABD patients do not have less mixity of symptom presentation nor are they more likely to show one pole of symptoms relative to the other or to be completely asymptomatic [22].

Regarding sex differences, women appeared to have higher scores on psychological anxiety, somatic anxiety, and hypochondriasis items regarding depressive mood symptoms [23]. When looking specifically into mania symptoms, women showed lower scores on the content item and higher scores on the insight item. Furthermore, female sex was associated with more psychiatric hospitalizations and fewer lifetime Substance Use Disorders (SUDs) [23]. Additionally, OABD women present with an earlier age of onset of BD than OABD men. Remarkably, in the replication study (W2) it was found that depression was associated with an older age but only among men [19]. In light of these results, sex should be considered a primary factor when analyzing OABD data. Clinically, it is crucial to take into account the differences between sexes when developing treatment strategies. Understanding these differences can lead to more effective and personalized treatment plans, improving patient outcomes and overall care.

The division of BD in BD-I and BD-II is a highly debated topic. Also in GAGE-BD, differences between these groups have been observed, with BD-I patients more often showing a history of psychiatric hospitalizations and a higher current use of antipsychotics [24]. BD-II patients more often had a late onset (> 50 years). Also, BD-II patients had more severe current depression. BD-I and BD-II groups were not different in terms of general functioning, cognitive performance, and somatic burden. These findings suggest that despite possible clinical differences, BD subtype does not influence overall functioning or physical health in older age [24].

Differences between early-onset (EOBD) and late-onset groups (LOBD) have been studied in GAGE-BD. Early-onset has been defined as BD onset before the age of 40, whereas late-onset refers to those patients who experience their first episode after the age of 40 [25]. Analysis of GAGE-BD data revealed no differences between the two groups in terms of depression, mania, or psychosocial functioning outcomes. However, endocrine comorbidities were more common in the LOBD group. The modest differences between the two groups indicate a shared biological mechanism underlying LOBD and EOBD and suggest that these groups do not represent a different subtype [25]. These results confirm the position in the debate that both groups truly share the same diagnostic entity but differ in the age of debut of the disease, rather then they should be conceptualized as two different clinical entities with distinct etiologies, but exhibit such a similar “phenotype” that both are categorized under the same diagnostic umbrella.

### Physical Health, Comorbidity, and Cognition

Considering somatic comorbidities in OABD is essential for understanding its impact on disease management and course. Previous studies have shown that the physical health burden is greater for BD than for the general population [26] and these comorbidities could worsen in severity and number with advancing age. Somatic comorbidity in the GAGE-BD sample is highly prevalent, particularly cardiovascular disease, seen in nearly half of individuals with OABD [18], with the oldest individuals having the highest burden of physical comorbidities [27]. When comparing OABD individuals to the general population, a faster accumulation of chronic physical diseases and a faster decline in health perception were observed. The observed differences could partly be attributed to baseline differences in psychosocial, lifestyle, and health behavior factors [28].

Importantly, sex differences have been found, where GAGE-BD findings from W1 showed that physical morbidities affect more women than men with OABD, with significant differences found for diseases affecting the gastrointestinal, musculoskeletal, and endocrinological systems [29]. Physical morbidities were more frequent in OABD men compared to men without OABD, particularly for cardiovascular, renal, and endocrine diseases, while gastrointestinal disorders were less prevalent. These findings were replicated in W2, and again it was found that women had more gastrointestinal genitourinary, musculoskeletal, and endocrine comorbidities than men with OABD, when age, education, smoking history, and study site were controlled [16]. These replicated GAGE-BD findings indicate that OABD presents more physical morbidities than matched comparison participants, and that this health burden is significantly greater among women. Long-term pharmacological treatment may be related to comorbidities in OABD, possibly due to selected drug-related adverse effects. Notably, among lithium users, there is a lower prevalence of cardiovascular diseases [30].

Another integral aspect of the OABD phenotype is cognition. GAGE-BD findings showed that there was no significant difference in overall cognitive functioning, between OABD and HC, while better global cognition was associated with working status in OABD [31]. Within OABD, better cognitive scores were associated with fewer manic symptoms, higher education and better functioning. Nonetheless, a previous meta-analysis that included a large number of cognitive domains, identified worse cognitive performance in OABD compared to healthy controls, with large and moderate effects in many cognitive domains [32]. These contradictory results among studies may stem from a lack of data harmonization and varying methods of analyzing cognitive function, whether through global scores or comprehensive assessment across cognitive domains. Standardizing or harmonizing data collection could provide more consistent findings and enhance our understanding of cognitive outcomes in diverse populations. Additionally, refining assessment protocols to capture nuanced cognitive abilities within specific domains may uncover subtleties missed by broader scoring approaches.

### Daily Functioning in OABD

Psychosocial functioning in OABD presents distinct characteristics and may predominantly affect different areas compared to younger adults. Cognitive decline, physical comorbidities, and social isolation are more prevalent, leading to greater impairments in daily living activities and overall independence. Additionally, older adults may face distinct challenges in areas such as mobility, self-care, and maintaining social networks, which are less prominent in younger populations.

Clinical BD symptoms significantly impact daily functioning across many everyday areas. Data from GAGE-BD W1 identified a higher association between more depressive symptoms with worse functioning [17, 18]. The replicated sample also confirmed the original results that functioning is relatively stable across adulthood and that worse BD symptom severity levels, but not somatic comorbidities, are associated with worse functioning in OABD, regardless of sex. Also, age moderated the relationship of depression severity with functioning, but in the opposite direction. So, in W1, older age had a stronger relationship with functioning, whereas in W2, younger age showed a stronger relationship with functioning. In the combined sample these seemed to cancel each other out [19]. More somatic comorbidities were not associated with reduced functioning in W1 [18], and this lack of an association between somatic comorbidities and functioning was also replicated with W2 analysis [19].

With respect to other elements of functional status, it was also found that lower educational attainment, older age, more severe depressive symptoms, more prior psychiatric hospitalizations, and more comorbidity (particularly the dual comorbidity of having substance use disorder and anxiety) were associated with greater odds of unemployment. In addition, a significant relationship between gender, BD age of onset, manic symptoms, and employment status was not found [33].

Taken together, effective management of OABD requires not only addressing clinical symptoms but also implementing strategies to enhance psychosocial support and optimize functioning, thereby improving the overall prognosis and quality of life for affected individuals. To help with measurement-based treatment planning and care, the FAST-O [34] is a recommended scale to measure daily functioning in OABD [35] and it has been translated in various languages (see gage-bd.org).

### Pharmacological Treatment

While cross-sectional analysis cannot support conclusions with respect to causality, lithium users in GAGE-BD had higher levels of education, were less likely to have family history of psychiatric disorders, or a personal history of psychiatric comorbidities [30]. They also had lower mean scores in depression severity scales and lower frequency of severe forms of depression, in addition to better global cognitive and functional state. Also, participants in the lithium-treated group were less likely to be prescribed antipsychotic drugs. As well, cardiovascular comorbidities were less frequent among lithium users. However, there may be a bias in lithium-treated patients, as lithium is often prescribed to individuals with specific characteristics that ensure strict adherence to treatment. Consequently, these patients are more likely to adopt healthier strategies to preserve their health, maintain mood stability, achieve improved clinical outcomes, and thus enhance their overall prognosis.

Also, antipsychotics-users (APS) and non-antipsychotic-users (no APS) were compared within GAGE-BD [20]. Most demographic and clinical variables were the same among OABD prescribed vs. not prescribed APS and there were no differences in somatic burden between the APS and no APS groups. However, individuals on APS were younger, less likely to be employed, less on lithium, and had more psychiatric hospitalizations possibly suggesting APS are used in those who have more severe BD.

## Future Directions

### What Can Clinicians Learn from GAGE-BD?

Cumulative analysis from the GAGE-BD data suggests OABD is a complex group in which multiple aspects of the disorder are interrelated. Our findings urgently call for the use of integrated care in BD. Understanding specific elements of clinical presentation patterns in OABD is crucial for enhancing prevention strategies, optimizing treatments, and developing more personalized approaches. By recognizing how symptoms and additional impairments manifest differently in older adults compared to younger populations, clinicians can tailor interventions more effectively. Moreover, understanding these differences allows for the implementation of preventive measures that address specific vulnerabilities associated with aging, ultimately promoting better long-term management of OABD. It should not be assumed or normalized that OABD patients will display poor outcomes due to their multiple conditions; instead, this can be optimized by integrating appropriate pharmacological, psychosocial, and psychological strategies improve health and prognosis.

### Future Plans Within GAGE-BD

Thus far, the GAGE-BD has compiled a large and unique dataset specific to OABD that has yielded new information and findings that can inform clinical care and a future research agenda. However, there are limitations to interpretations that can be drawn given methodological features of the existing GAGE-BD sample and structure. The GAGE-BD team is in the process of improving methodological rigor in multiple areas, including geographical representativeness and data quality, data sources with respect to original purpose and support/sponsorship, comparison groups and diversity of the GAGE-BD leadership team to plan future strategy. With respect to geographical representation, newly acquire W3 data has required a minimum OABD sample size, the use of standardized measures on key outcome variables (for example, use of validated measures for mood symptom severity) and an available data dictionary that will facilitate aggregate data analysis. Up until now, the GAGE-BD dataset has not included research samples collected by the pharmaceutical industry (Pharma), which are an important data source in drug development and application. The GAGE-BD team has convened a Pharma Corporate Council (CC) to develop a roadmap strategy to guide engagement of Pharma partners in future iterations of analysis strategy and data acquisition. While the GAGE-BD sample has focused on OABD, the team strongly encourages the contribution of healthy control and older adults with other chronic mental illness cohorts (especially those with unipolar depression) as comparison sub-samples. To help expand the representativeness of the GAGE-BD global Steering Committee, GAGE-BD now includes input from CC members to provide non-branded input, the inclusion of early and mid-career investigator involvement to help grow the pipeline of OABD-focused research and individuals with lived experience of BD to add context and understanding of research needs and how findings might best be applied to advance care of people with BD across the life-span. Additional information about GAGE-BD is available on the project website (https://gage-bd.org/).

### Future Research Directions

Given demographic trends and growing awareness that OABD is not a niche or “special population” irrelevant to general clinical practice, there is need for future research that can inform care. Big data approaches such as the GAGE-BD project partially help to address the paucity of research on OABD in general. However, secondary or post-hoc analyses are not powered to adequately test novel care approaches and prospective clinical trials are needed to rigorously examine both biological and psychosocial therapies. A recent set of recommendations provide a battery of outcomes evaluations that can be considered in prospective studies [36]. A final caveat is that convenience samples of older adults, such as the standard interventional clinical trial, tends to enroll a “healthy survivor” cohort. Longitudinal, population-based studies that can examine factors relevant to resilience, survivorship and recovery are needed to fully understand the biological and psychosocial drivers of health outcomes among people with bipolar disorder across the life-span.

## Conclusions

GAGE-BD is the largest cohort focusing on aging with BD including approximately 3500 people with BD and 1400 comparison participants without BD. Despite the limitations inherent to an archival dataset with multiple studies and sites GAGE-BD has provided novel insights including that BD does not fade with age, and both manic and depressive symptoms are strong drivers of functional status. Subtyping BD-I and BD-II or early-onset and late-onset of the OABD sample did not reveal relevant clinical distinctions, in terms of daily functioning. Physical comorbidities are frequent in OABD and especially pronounced in women. Cognitive and physical comorbidities, together with changes in social network and clinical BD symptoms can lead to impaired daily functioning in OABD. Moving forward, it is critical to fill the remaining gaps in knowledge regarding assessment and care for people with OABD. While datasets such as GAGE-BD are helpful, future global efforts using diverse methods will be needed to more fully understand and develop strategies that can help advance care for this rapidly growing and vulnerable population.

## Key References

- Sajatovic M, Eyler LT, Rej S, et al. The Global Aging & Geriatric Experiments in Bipolar Disorder Database (GAGE-BD) project: Understanding older-age bipolar disorder by combining multiple datasets. Bipolar Disord. 2019;21:642-9. **This study explains the aims and methods of the GAGE-BD project**.
- Teixeira AL, Almeida OP, Lavin P, et al. Physical comorbidities of older age bipolar disorder (OABD) patients: A global replication analysis of prevalence and sex differences. Gen Hosp Psychiatry. 2024;90:6–11. **This study is a GAGE-BD replication study and showed that OABD present more physical morbidities than matched controls**,** and that this health burden is significantly greater among women.**
- Sajatovic M, Dols A, Rej S, et al. Bipolar symptoms, somatic burden, and functioning in older-age bipolar disorder: Analyses from the Global Aging & Geriatric Experiments in Bipolar Disorder Database project. Bipolar Disord. 2022;24:195–206. **This study shows the results of the first wave of the Global Aging & Geriatric Experiments in Bipolar Disorder Database (GAGE-BD) project. Findings suggest an attenuation of BD symptoms in OABD**,** despite extensive somatic burden. Depressive symptom severity was strongly associated with worse functioning.**
- Sajatovic M, Rej S, Almeida OP, et al. Bipolar symptoms, somatic burden and functioning in older-age bipolar disorder: A replication study from the global aging & geriatric experiments in bipolar disorder database (GAGE‐BD) project. Int J Geriatr Psychiatry. 2024;39: e6057. **This is a replication study of the GAGE-BD project and shows results of both wave 1 and wave 2. It was found that an older age was associated with less severe mania and more somatic burden**,** but that there was no association of depression with age. Worse BD symptoms were also associated with worse functioning.**
- Blanken MA, Oudega ML, Almeida OP, et al. Sex Differences Among Older Adults With Bipolar Disorder: Results From the Global Aging & Geriatric Experiments in Bipolar Disorder (GAGE-BD) Project. Am J Geriatr Psychiatry. 2024;32:326–338. **This GAGE-BD study showed that sex differences are present in OABD**,** with older aged women experiencing a more severe course of BD**,** with higher rates of psychiatric hospitalization.**
- Beunders AJ, Klaus F, Kok AA, et al. Bipolar I and bipolar II subtypes in older age: Results from the Global Aging and Geriatric Experiments in Bipolar Disorder (GAGE-BD) project. Bipolar Disord. 2023;25:43–55. **This GAGE-BD study showed that BD-I and BD-II patients did not differ in terms of general functioning**,** cognitive impairment or somatic burden.**
- Almeida OP, Dols A, Blanken MA. Physical health burden among older men and women with bipolar disorder: results from the Gage-Bd Collaboration. Am J Geriatr Psychiatry. 2022;30:727–732. **These GAGE-BD findings showed that extensive physical burden is present in OABD patients. Within the OABD group**,** women were more affected than men.**

## Electronic Supplementary Material

Below is the link to the electronic supplementary material.

Supplementary Material 1

## Data Availability

No datasets were generated or analysed during the current study.
