# Supplementary material for: Global Aging & Geriatric Experiments in Bipolar Disorder (GAGE-BD): Building a Global Consortium Benefiting People with Bipolar Disorder in Later Life
Source: Curr Psychiatry Rep. 2025 Mar 11;27(5):231–8. doi: 10.1007/s11920-025-01593-w (PMC12003440; doi:10.1007/s11920-025-01593-w)
Supplement: Supplementary file 1 — Supplementary Material 1 [file 11920_2025_1593_MOESM1_ESM.docx]

**GAGE-BD FULL WAVE-1 & 2 SUPPLEMENTAL DATA**

**Supplemental table 1.   Meta-data for the contributing studies.**

| **Full name of study** | | **Data Wave** | **Study cohort acronym** | **Site** | **Study design** | **Total N of study in dataset (BD and non-BD)** | **Location** |
| --- | --- | --- | --- | --- | --- | --- | --- |
| Atorvastatin for the Treatment of Lithium-Induced Nephrogenic Diabetes Insipidus: A Randomized Controlled Trial | | 1 | Atorvastatin | Lady Davis Institute | RCT | 13 | Quebec, Canada (NORTH AMERICA) |
| Treatment Adherence Enhancement in Bipolar Disorder | | 1 | CAE | Case Western Reserve University (multi-site study) | RCT | 184 | Ohio, USA (NORTH AMERICA) |
| Cognition in Bipolar Disorder XR | | 1 | CiBS_XR | GGZ inGeest | Observational | 197 | Amsterdam, Netherlands (EUROPE AND CENTRAL ASIA) |
| Cognition in Euthymic Older Adults with Bipolar Disorder | | 1 | Cog-BD | Center for Addiction & Mental Health | Observational | 48 | Ontario, Canada (NORTH AMERICA) |
| Dutch Older Bipolar cohort, wave 1 | | 1 | DOBi1 | GGZ inGeest | Observational | 101 | Amsterdam, Netherlands (EUROPE AND CENTRAL ASIA) |
| Dutch Older Bipolar cohort, wave 2 | | 1 | DOBi2 | GGZ inGeest | Observational | 69 | Amsterdam, Netherlands (EUROPE AND CENTRAL ASIA) |
| Open-label, Prospective Trial of Lamotrigine for Symptoms of Geriatric Bipolar Depression | | 1 | GERI-SAD | Case Western Reserve University | Prospective  uncontrolled  intervention with Lamotrigine | 53 | Ohio, USA (NORTH AMERICA) |
| Geriatric Psychiatry Mood Disorders Research Database | | 1 | GMDD | McLean Hospital | Observational | 127 | Massachusetts, USA (NORTH AMERICA) |
| Health in Men Study | | 1 | HIMS | University of Western Australia | Observational | 68 | Perth, Australia (EAST ASIA AND PACIFIC) |
| Dynamic Inflammatory and Mood Predictors of Cognitive Aging in Bipolar Disorder | | 1 | Inflammaging | University of California San  Diego | Observational | 168 | California, USA (NORTH AMERICA) |
| The McGill Geriatric Lithium-Induced Diabetes Insipidus Clinical Study | | 1 | McGLIDICS | McGill University | Observational | 100 | Quebec, Canada (NORTH AMERICA) |
| Cognitive Impairment and dementia in late life bipolar disorder | | 1 | OABD_S | University of Sao Paolo | Observational | 144 | São Paulo, Brazil (LATIN AMERICA AND THE CARIBBEAN) |
| Asenapine in the Treatment of Older Adults With Bipolar Disorder | | 1 | OPT-BD | Case Western Reserve University | Prospective  uncontrolled  intervention with Asenapine | 15 | Ohio, USA (NORTH AMERICA) |
| Effects of medication and medical morbidity on brain and cognition of old bipolar patients | | 1 | TMU 1 | Taipei Medical University | Observational | 56 | Taipei, Taiwan (EAST ASIA AND PACIFIC) |
| Effects of medication and medical morbidity on brain and cognition of old bipolar patients | | 1 | TMU2 | Taipei Medical University | Observational | 42 | Taipei, Taiwan (EAST ASIA AND PACIFIC) |
| University of Barcelona Bipolar  Disorder Program Cohort | | 1 | UBBDPC | University of Barcelona | Observational | 136 | Barcelona, Spain (EUROPE AND CENTRAL ASIA) |
| The Effect of Bipolar Disorder and its Comorbidities on Cognition in Older Adults | | 1 | UPMC | University of Pittsburgh Medical Center | Observational | 149 | Pennsylvania, USA (NORTH AMERICA) |
| Mood Disorders Research Program  Database | | 1 | Yale | Yale School of Medicine | Observational | 88 | Connecticut, USA (NORTH AMERICA) |
| Ziprasidone switching in response to  adherence in psychotropic-related  weight gain concerns among patients  with bipolar disorder | | 1 | ZIP-AD | Case Western Reserve University | Prospective  uncontrolled  intervention with Ziprasidone | 30 | Ohio, USA (NORTH AMERICA) |
| **Full name of study** | |  | **Study cohort acronym** | **Site** | **Study design** | **Total N** |  |
| The Maritime Bipolar Registry: Outcome of Bipolar Disorder in a Community-Treated Sample \|  Phenotypic and Molecular Heterogeneity of Bipolar Disorder | | 2 | Alda_Hajek_1 | Halifax | Observational | 565 | Nova Scotia, Canada (NORTH AMERICA) |
| Several studies as described in “*Midline Brain Abnormalities Across Psychotic and Mood Disorders*"† | | 2 | Barcelona(FIDMAG) | Hospital Clínic Barcelona / FIDMAG Germanes Hospitalaries | Observational | 119 | Barcelona, Spain (EUROPE AND CENTRAL ASIA) |
| Enhancing adherence and outcomes in bipolar disorder with Abilify Maintena + a targeted behavioral approach to promote sustained adherence and behavioral change | | 2 | BD-CAEL | CWRU | Prospective trial with Maintena | 30 | Ohio, USA (NORTH AMERICA) |
| University of Barcelona Bipolar Disorder Program Cohort | | 2 | UBBDPC | University of Barcelona | Observational | 52 | Barcelona, Spain (EUROPE AND CENTRAL ASIA) |
| Assessment of clinical conditions and biomarkers related to psychiatric outcomes and cognition in patients with bipolar disorder | | 2 | Belo Horizonte(Bipolar Cordis Est) | UFMG | Observational | 186 | Belo Horizonte, Brazil (LATIN AMERICA AND THE CARIBBEAN) |
| Acute Pharmacotherapy of Late-Life Mania | | 2 | GERI-BD | CWRU | RCT | 224 | Ohio, USA (NORTH AMERICA) |
| Improving Medication Adherence in Hypertensive Individuals with Bipolar Disorder (iTAB-CV) - Phase 2 | | 2 | iTAB-CVPilot | CWRU | Prospective cohort design | 38 | Ohio, USA (NORTH AMERICA) |
| Mazhar Osman Mood Clinic | | 2 | Konya | Konya - Selçuk University Mazhar Osman Mood Clinic | Observational | 29 | Konya, Türkiye (EUROPE AND CENTRAL ASIA) |
| Mood and Metabolism Program Database | | 2 | M&M | Halifax | Clinical database | 217 | Nova Scotia, Canada (NORTH AMERICA) |
| The Global Aging and geriatric experiments in Bipolar Disorder Database project. | | 2 | FNPHLag | Federal Neuropsychiatric Hospital/  McMaster University | Observational | 50 | Lagos, Nigeria (SUB-SAHARAN AFRICA) |
| Early diagnosis, treatment and prevention of mood disorders targeting the activated inflammatory response system- Bipolar patients cohort | | 2 | MOODINFLAME | University of Groningen | Observational | 105 | Groningen, Netherlands (EUROPE AND CENTRAL ASIA) |
| Bipolar Disorder Research Program:  Cognitive-Behavioral Rehabilitation Versus Treatment as Usual for Bipolar Patients aka "CBT"                                              &  C Monohydrate as Adjuvant Therapy for Bipolar Depression  aka "Creatine " | | 2 | PROMAN | University of Sao Paulo -PROMAN | RCT | 38 | São Paulo, Brazil (LATIN AMERICA AND THE CARIBBEAN) |
| Targeted Training in Illness Management (TTIM) for individuals with Serious Mental Illness (SMI) and Diabetes Mellitus (DM) Study Protocol | | 2 | TTIM | CWRU | RCT | 200 | Ohio, USA (NORTH AMERICA) |
| UTHealth Center of Excellence on Mood Disorders | | 2 | UTH_Houston | Houston | Observational | 123 | Texas, USA (NORTH AMERICA) |
| UTHealth Center of Excellence on Mood Disorders | | 2 | UTH_SanAntonio | Houston | Observational | 450 | Texas, USA (NORTH AMERICA) |
| UTHealth Center of Excellence on Mood Disorders | | 2 | UTH_UNC | Houston | Observational | 136 | Texas, USA (NORTH AMERICA) |
| Structural and Functional Brain Aging in Bipolar Disorder | | 2 | VA_BAI | Veterans Affairs San Diego Healthcare System | Observational | 126 | California, USA (NORTH AMERICA) |
| A Prospective 4-Year Naturalistic Follow-Up of Treatment and Outcome of 300 Bipolar I and II Patients | | 2 | Vienna(SFU) (Neunkirchen Cohort) | Neunkirchen | Observational Consecutive | 300 | Vienna, Austria (EUROPE AND CENTRAL ASIA) |
| Valencia Outpatient Unit. Cognitive and Social Functioning in Bipolar Disorder | | 2 | VOCS_BD | La Ribera University Hospital. Alzira, Valencia | Observational | 73 | Valencia, Spain (EUROPE AND CENTRAL ASIA) |
| **Total N** |  | | |  |  | **4822** |  |

**Notes:** RCT = Randomized controlled trial

† Landin-Romero, R., Amann, B. L., Sarró, S., Guerrero-Pedraza, A., Vicens, V., Rodriguez-Cano, E., Vieta, E., Salvador, R., Pomarol-Clotet, E., & Radua, J. (2016). Midline Brain Abnormalities Across Psychotic and Mood Disorders. Schizophrenia bulletin, 42(1), 229–238. https://doi.org/10.1093/schbul/sbv097

**Supplemental Table 2.**
**Inclusion and exclusion criteria for each contributing study**

| **Study cohort acronym** | **Inclusion criteria** | **Exclusion criteria** | |  |
| --- | --- | --- | --- | --- |
| Atorvastatin | 1. Individuals of 18 years of age or older (including patients 18-64 and 65+, with no maximum age limit) 2. Individuals with bipolar disorder in any phase of illness: euthymic, depressed, or hypomanic. Patients were recruited from the outpatient bipolar and geriatric psychiatry clinics 3. Able and willing to give informed consent 4. Chronic and current lithium users (at least 2 months of Lithium use) 5. Stable dose of lithium for the past 2 months.  - Patients taking any lithium level will be included 6. In the original study, patients with any psychiatric diagnosis were included, and had either bipolar disorder (n=54) or unipolar depression (n=6). Only patients with bipolar disorder who re-consented to data sharing with GAGE-BD were included in the current GAGE-BD analysis. 7. Patients were included in the atorvastatin trial if they had partial or complete nephrogenic diabetes insipidus (NDI) - defined as a 10-hour water restriction urine osmolality (UOsm) ≤300mOsm/Kg | 1. Patients with statin use within 6 weeks prior to the study  2. Patients with a past history of severe adverse reaction to statins  3. Patients with a baseline Low Density Lipoprotein (LDL) level <1.5  4. Relative contraindications to statin use 42: pregnancy or lactation, concurrent use of fibrates, heavy ethanol consumption (>50 units/week)  5. Incapacity to consent  6. Deemed by the treating physician to have a severe cognitive or behavioural disturbance such as acute delirium or moderate-severe DSM5 Neurocognitive Disorder (dementia), preventing their ability to complete safely the study questionnaire and/or to provide blood and urine test. | |  |
| CAE | 1. Subjects must have type I or type II Bipolar Disorder (BD) as confirmed by the Structured Clinical Interview for DSM-IV Axis I Disorders (SCID) 2. Have had BD for at least two years duration  3. Have received treatment with at least one evidence-based medication to stabilize mood for at least six months (lithium, anticonvulsant, or antipsychotic mood stabilizer)  4. Either 20% or more nonadherent with current BD medication treatment (i.e. lithium, anticonvulsant, or antipsychotic mood stabilizer)  5. Be able to participate in psychiatric interviews | 1. Unable or unwilling to participate in psychiatric  interviews. This will include individuals, who may be  too psychotic to participate in interviews/rating scales 2. Unable or unwilling to give written, informed consent to study participation  3. Children under the age of 18  4. Individuals at high risk for suicide who cannot be safely managed in their current treatment setting | |  |
| CiBS_XR | 1. 60+ years old  2. Diagnosis of bipolar I or bipolar II  3. Outpatients 4. Euthymic for at least 3 weeks as assessed by patient's psychiatrist  5. No history of ECT  Control group:  1. 60+ years old  2. No current or lifetime psychiatric illness or addict | 1. Not euthymic as assessed by YMRS, CESD  2. History of ECT  3. Alcohol dependency or substance abuse  4. Dementia  Controls:  1. History of psychiatric illness or addiction  2. Recent memory complaint | |  |
| Cog-BD | 1. Age 50 years and above  2. Meets DSM-IV TR criteria for a current diagnosis of Bipolar I or II Disorder  3. Willingness and ability to speak English  4. Willingness to provide informed consent  5. Corrected visual ability that enables reading of newspaper headlines and hearing capacity that is adequate to respond to a raised conversational voice.  6. At time of assessment they should be clinically euthymic for  four weeks preceding study entry, with both HRSD-17 and YMRS scores of 10 or less at time of assessment (The criteria are selected  to capture bipolar disorder across the older adults. We are interested in bipolar I and II disorder to capture bipolar illness that would generalize to "real-world," clinical practice. Although there are no set criteria for designating "acceptable" euthymia by HRSD-17 and YMRS, there is growing consensus among geriatric psychiatrists that scores 10 or less on these instruments are indicated to minimize acute impact of mood symptoms when performing NP testing). | 1. Does not meet criteria for any type of dementia or  other neurological disorder affecting the central nervous system (for example, multiple sclerosis, history of traumatic brain injury, cerebrovascular disease)  2. No history of schizophrenia, schizoaffective or other psychotic disorders  3. No alcohol or other drug abuse/dependence within 6 months of testing  4. No Electroconvulsive Therapy (ECT) within 6 months of testing. | |  |
| DOBi1 | 1. Age ≥ 60 years 2. Diagnostic procedure (MINI) indicates BD-I, BD-II, or BD-NOS  3. Willing to give consent (some consented to chart review plus structured interview, others consented to chart review only) | 1. Dementia  2. Intellectual disability (IQ<70)  3. Language barrier  4. Poor cognition (measured by Mini Mental State Examination; MMSE <18  5. Insufficiently stable psychiatric condition | |  |
| DOBi2 | 1. Age ≥ 50 years 2. Diagnostic procedure (MINI) indicates BD-I, BD-II, or BD-NOS  3. Willing to give consent (some consented to chart review plus structured interview, others consented to chart review only) | 1. Dementia  2. Intellectual disability (IQ<70)  3. Language barrier  4. Poor cognition (measured by Mini Mental State Examination; MMSE <18)  5. Insufficiently stable psychiatric condition | |  |
| GERI-SAD | 1. Age > 60 Years  2. BP Disorder-I or II: Depressive episode (DSM -IV-TR; SCID-I/P)  3. HAM-D > 18 (GRIDHAM-D 24-item version)  4. Availability of an informant is encouraged but not required for study participation. | 1. Chronic psychotic conditions, ie. schizophrenia, schizoaffective disorder, delusional disorder 2. Contraindication to lamotrigine (Physician interview, medical assessment)  3. Documented history of intolerance to lamotrigine  4. Patients who have previously failed to respond to at  least 12 weeks of treatment with lamotrigine  5. Active substance dependence (SCID-I/P) or substance-related safety issues or PI concerns  6. Mood Disorder Due to a General Medical Condition or Treatment (Physician interview)  7. Rapid cycling (Physician interview): As defined in DSM-IV: At least 4 episodes of mood disturbance in the previous 12 months that meet criteria for a Major Depressive, Manic, Mixed or Hypomanic Episode. Episodes are distinguished either by partial or full remission for at least 2 months or by a switch to an episode of opposite polarity  8. Dementia (by DSM-IV or brain degenerative diseases; Physician interview)  9. Inability to communicate in English (i.e., interview cannot be conducted without an interpreter; subject  largely unable to understand questions and cannot respond in English)  10. Clinically significant sensory impairment (i.e., cannot see well enough to read consent or visually presented material; cannot hear well enough to  cooperate with interview; Physician interview)  11. Recent history of cardiovascular, peripheral vascular events or stroke 12. High risk for suicide (e.g., active SI or current intent or plan)  13. Inpatient status. | |  |
| HIMS | 1. Australian men aged 65–85 living in the Perth metropolitan region during 1996–1998 using the Australian Electoral Roll  2. Eligible for cardiovascular health screening  3. BD diagnosis based on ICD-10 health service use codes | 1. Residing outside of study areas  2. Unable or unwilling to participate in or complete cardiovascular screening  3. < age 65 years | |  |
| GMDD | Participants: 1. Diagnosis of Major Depressive Disorder, Depression NOS, Dysthymia, Bipolar Disorder, Type I or II, Bipolar Depression  NOS, or any other diagnosis of a mood disorder  2. Ability to provide Informed Consent  3. Age 55-89, inclusive  Control subjects:  1. Ability to provide informed consent  2. Age 55-89, inclusive | Participants: 1. Inability to complete diagnostic assessment (not including intelligence testing)  2. Inability to speak English  3. Active substance abuse or dependence (other than caffeine or nicotine) within the last month as  determined by SCID DSM-IV, MINI, or clinical  judgment.  Control subjects:  1. Inability to speak English  2. Inability to complete diagnostic assessment (not including intelligence testing)  3. History or current diagnosis of the following psychiatric illnesses: any organic mental disorder (including dementia), schizophrenia, schizoaffective disorder, delusional disorder, psychotic disorder not  otherwise specified, unipolar major depressive disorder, bipolar disorder, patients with substance abuse or dependence disorders, including alcohol  4. Serious or unstable medical illness, including cardiovascular, hepatic, renal, respiratory, endocrine,  neurologic or hematologic disease | |  |
| Inflammaging | 1. Diagnosis of Bipolar I or II Disorder by DSM-IV criteria  2. Age 25-60 years, currently outpatient, proficient in English 3. Capable of providing informed consent. | 1. Acute medical illness (e.g., cold, flu, bacterial infection, heart failure, cancer) or pregnancy  2. Recent (<6 weeks) vaccination  3. History of neurological disorder (e.g., dementia, seizures, Parkinson's, stroke) or head trauma with unconsciousness > 15 minutes  4. Diagnosis of substance abuse within the last 3 months or dependence within the last 6 months  5. History of radiation or chemotherapy treatment,  uncontrolled diabetes or hypertension, sensory limitations including vision uncorrectable to 20/40, conservatorized, color blindness or hearing loss that interferes with assessment, chronic pain that necessitates treatment with nonsteroidal anti-inflammatory drugs or prescription painkillers that would affect blood-based markers of inflammation. | |  |
| McGLIDICS | Current or past exposure to lithium | No exclusion criteria | |  |
| OABD_S | Patient group:  1. Older adults (60 years or more) with major affective disorders, i.e. late-life bipolar disorders; or geriatric depression  Comparison groups:  1. Minor Neurocognitive Disorder (DSM-V) or equivalent diagnosis of MCI according to Mayo Clinic criteria; these  subjects will be subdivided according to their profile of  cognitive deficits (amnestic; non-amnestic MCI) and the identification of the 'AD-signature' in the CSF as indicated by the  concentrations of AD-related biomarkers (low amyloid-beta and high total Tau and phospho-Tau)  2. Major Neurocognitive Disorder due to AD (DSM-V), sub-grouped according to the age of onset of dementia (i.e., early- or  late-onset AD)  3. Healthy older adults with normal cognitive function (controls). | 1. Illiteracy  2. Diagnosis of other major DSM-IV Axis I disorders  3. Presence of any acute or major unstable medical illness or organic brain syndromes including dementia (other than AD)  4. Current use of medications to treat medical comorbidities that could possibly affect biological outcome variables (such as non-steroidal  anti-inflammatories, insulin or other anti-diabetic drugs)  5. Withdrawal or refusal to sign the informed consent (previously approved by the local ethics committee). | |  |
| OPT-BD | 1. Subjects must have type I Bipolar disorder by DSM-IV  criteria confirmed on the Mini Neuropsychiatric Interview (MINI)  2. Subjects must be age 60 or older  3. Subjects must have suboptimal response to current psychotropic management including at least one of the following:  a. Behaviors and symptoms of irritability, agitation, mood lability or diminished ability to interact with others in their place of  residence b. Diminished ability to take care of basic personal needs in their place of residence due to symptoms of BD | 1. History of intolerance or resistance to Asenapine  2. Clinical diagnosis of dementia or Mini-mental state (MMSE) < 24  3. History of TIA, stroke or MI within the past 12 months  4. Medical illness that is the clear, underlying etiology of BD  5. Unstable medical illness or condition including prolonged QT interval, which in the opinion of the study investigators, is likely to affect the outcome of the study or the subject's safety  6. DSM-IV substance dependence (except nicotine or caffeine) within the past 3 months.  7. Rapid cycling BD defined as 4 or more discrete mood episodes within the previous 12 months.  8. At high risk for self-harm or suicide | |  |
| TMU 1 & TMU2 | 1. Age 60 years and over 2. Having a final diagnosis of DSM-IV bipolar I disorder 3. Having at least one psychiatric admission to TCPC or TMUH before the start of the study. | 1. Patients with comorbid dementia due to other general medical conditions, neurological diseases, and active substance abuse were excluded  2. Must have achieved symptomatic remission prior to study start. | |  |
| UBBDPC | 1. Being aged 18 or older 2. Presenting a diagnosis of bipolar disorder following DSM-IV-TR criteria  3. Being outpatient at the moment of the assessment | 1. History of intellectual disability 2. Any medical condition that could interfere in the assessment procedure | |  |
| UPMC | 1. Age ≥ 50 years 2. Clinical euthymia for four weeks preceding neurocognitive assessment with scores of ≤ 10 on both the 17-item Hamilton  Rating Scale for Depression (HRSD) and the Young Mania  Rating Scale (YMRS) at the time of assessment  3. Ability to comprehend and speak English fluently  4. Corrected visual ability to read newspaper headlines  5. Hearing capacity adequate to respond to a raised  conversational voice | 1. History of dementia or neurologic disorder affecting the central nervous system (e.g., Parkinson's  disease, traumatic brain injury, or multiple sclerosis)  2. Electroconvulsive therapy within the past six months  3. Substance abuse or dependence within the past 12 months.  4. For this report, we focused on subjects who had completed both neuroimaging and neurocognitive  assessment. | |  |
| Yale | 1. BP 1 or 2  2. No contraindications to MRI scanning (see below)  4. No recent substance abuse/dependence | For the majority of the subjects, exclusion  criteria were:  1. Any contraindications for magnetic resonance imaging (MRI)  2. Intellectual disability (IQ<70)  3. Pregnancy  4. Significant or unstable neurological illness including structural brain disease or epilepsy that could have major effects on the brain  5. History of loss of consciousness for more than 5 minutes  6. Significant or unstable medical illness that could affect the brain e.g. insulin dependent diabetes | |  |
| ZIP-AD | 1. Diagnosis of Type I or II BD for at least 6 months (confirmed with MINI)  2. On maintenance evidence-based treatment for BD (lithium, antipsychotic, anticonvulsant)  3. Have weight gain concerns that individual believes are related to BD medication treatment.  4. Sub-optimal adherence as measured by the Tablet Routines Questionnaire (TRQ) and which the patient feels is related to  weight gain concerns. TRQ threshold will be defined as missing 20% or more of prescribed BD treatment in last week or last month. This is consistent with methodologies in PIs previous BD adherence studies. | 1. Known resistance or intolerance to ziprasidone.  2. Medical contraindication to ziprasidone.  3. Individuals on ziprasidone immediately prior to study  enrollment.  4. Prior or current treatment with clozapine.  5. Diagnosis of eating disorder  6. Individuals whose sub-optimal adherence is related to inability to pay for BD medication treatment or inability to arrange transportation to BD treatment clinical visits  7. Concurrent medical condition or psychiatric illness, which in the opinion of the research psychiatrist,  would interfere with the patient's ability to participate in the trial.  8. Current substance dependence.  9. High risk of harm to self or others.  10. Female who is currently pregnant or breastfeeding. | |  |
| **WAVE 2:**  **Study cohort acronym** | **Inclusion criteria** | **Exclusion criteria** | |  |
| Alda_Hajek_1 | **Li neuroprotection study:**  *The BD patients (both Li and non-Li groups) had to have:*  1. A diagnosis of bipolar I or II disorder made by a psychiatrist using the SCID;  2. At least 10 years of illness;  3. A history of at least five episodes of illness (including manic, depressive, or mixed episodes);  4. Current Hamilton Depression Rating Scale, 17-item version (HAM-D-17) score < 7;  5.Current Young Mania Rating Scale (YMRS) score < 5;  6.Current Clinical Global Impressions Scale-Bipolar (CGI-BP) score < 3;  7.A period of euthymia for at least four months prior to scanning, as aside from state-related factors, patients in acute episodes may present with additional difficult to control confounding variables, including recent medication change or substance abuse. The non-Li group had to have less than three months of lifetime Li exposure, more than 24 months prior to the scanning. The Li group had to have a current Li treatment lasting a minimum of 24 months.  **Diabetes Study:**  The subjects with BD were required to  1. have the diagnosis of bipolar I or II disorder made by a psychiatrist; and  2. be at least 18 years of age. | **Li neuroprotection study:**  Individuals from any of the three groups were excluded if they met any of the magnetic resonance imaging (MRI) exclusion criteria or had any serious medical illness (e.g., brain injury, Cushings disease, or conditions treated with corticosteroids). Individuals with BD were excluded if they had:  1. More than one lifetime course of electroconvulsive therapy (ECT) or ECT in the previous 12 months;  2. Comorbid psychiatric disorders, and ⁄ or personality disorder;  3. Active substance abuse in the previous 12 months;  4. Significant change in their medication in the previous three months; or  5. Current psychotic features or acute suicidality.  Individuals from the non-Li group were excluded if they had:  1.Li exposure < 2 years before the scanning; or  2.lifetime Li exposure of more than three months.  The neuropsychiatrically healthy individuals were excluded if they had a personal history of psychiatric disorders.  **Diabetes Study:**  The neuropsychiatrically healthy, euglycemic subjects were excluded if they had  1.a personal history of psychiatric disorders; or  2.T2DM.  Subjects from any group were excluded if they  1. met any magnetic resonance imaging (MRI) exclusion criteria;  2. suffered from substance abuse in the last 12 months;  3. had a history of  neurodegenerative disorders; or  4. cerebrovascular disease/stroke, as we were interested in the more subtle T2DM-related neuronal changes.  Patients were excluded if they had  1. the diagnosis of organic mood disorder;  2. mood disorder not otherwise specified; or  3. more than one lifetime course of electroconvulsive therapy or electroconvulsive therapy within the last 6 months. | |  |
| Barcelona(FIDMAG) | All patients with bipolar disorder were right-handed. All patients were diagnosed using DSM-IV and Research Diagnostic Criteria (RDC), based on a detailed clinical interview and review of case notes. | 1.Age younger than 18 or older than 65 years  2.History of neurological disease or brain trauma  3.Alcohol/substance abuse in the 12 months prior to participation  4.Non-right-handedness  5.Current IQ below the normal range (>70).  **Additional exclusion criteria for healthy controls were**  6.History of mental illness and/or treatment with psychotropic medication other than non-regular use of benzodiazepines or similar drugs for insomnia  7.If a first-degree relative had experienced symptoms consistent with major psychiatric disorder and/or had received any form of in-or outpatient psychiatric care. | |  |
| BD-CAEL | 1.Individuals age 18 and older with BD Type 1 or 2 as confirmed by the Mini International Psychiatric Inventory (MINI).  2.Known to have medication treatment adherence problems as identified by the Treatment Routines Questionnaire (TRQ, 20% or more missed medications in past week or past month)  3.Screening BPRS score of ≥ 36  4.Ability to be rated on psychiatric rating scales.  5.Willingness to take LAI  6.Currently in treatment or scheduled to receive treatment at a Community Mental Health Clinic (CMHC) or other clinical setting able to provide mental health care during and after study participation  7.Able to provide written, informed consent to study participation. | 1.Individuals on LAI immediately prior to study enrollment.  2.Individuals with known intolerance or resistance to either oral aripiprazole or LAI formulation of aripiprazole  3.Prior or current treatment with clozapine  4.Medical condition or illness, which in the opinion of the research psychiatrist, would interfere with the patient's ability to participate in the trial  5.Physical dependence on substances (alcohol or illicit drugs) likely to lead to withdrawal reaction during the course of the study in the clinical opinion of the treated research psychiatrist  6.Immediate risk of harm to self or others  7.Female who is currently pregnant or breastfeeding | |  |
| UBBDPC | 1. Being aged 18 or older  2. Presenting a diagnosis of bipolar disorder following DSM-IV-TR criteria  3. Being outpatient at the moment of the assessment | 1. History of intellectual disability  2. Any medical condition that could interfere in the assessment procedure | |  |
| Belo Horizonte (Bipolar Cordis Est) | 1.All subjects had to be 18 or older and to provide written informed consent.  2.All subjects were assessed with the Mini International Neuropsychiatric Interview (MINI-Plus), and bipolar disorder diagnosis was performed according to DSM-IV-TR criteria.  3.Patients were recruited at our outpatient clinic, and all have been followed there for at least 6 months to ensure the diagnosis of BD.  4.Controls were recruited in the community, and they should not have any current or past psychiatric diagnosis and family history of major psychiatric disorders.  5.BD 1 diagnosis | 1.Severe and/or unstable clinical comorbidities (e.g., chronic renal disease IV or V, congestive heart failure)  2.Use in the previous 4 weeks of any antibiotics, corticosteroids or immunosuppressants. | |  |
| GERI-BD | 1. Age greater than or equal to 60 years  2. Bipolar Disorder, Type I: Current Manic, Mixed or Hypomanic Episode as defined by the Diagnostic and Statistical Manual for Mental Disorders - Fourth Edition and as confirmed by Structured Clinical Interview for DSM-IV Disorders (SCID-P).  3. Young Mania Rating Scale (YMRS) total score greater than or equal to 18. | 1. Chronic psychotic conditions (i.e., schizophrenia, schizoaffective disorder).  2. Contraindication to study medications  3. Documented history of intolerance of LI, DV, lorazepam, or risperdone.  4. Patients who have failed to respond to at least 4 weeks of treatment with LI (greater than or equal to 0.4mEq/L) or VAL (greater than or equal to 40 ug/ml)  5. Active substance dependence or any substance-related safety issues or PI concerns.  6. Mood Disorder Due to a General Medical Condition or Treatment. Patients with mania associated with use of steroids, L-DOPA use, recent stroke, hyperthyroidism, porphyria, HIV infection, and connective tissue diseases, are excluded because the proposed interventions may be insufficient for their treatment.  7. Patients with rapid cycling BP disorder will be excluded, because these patients may require different treatments and should be studied separately.  8. Dementia and brain degenerative diseases.  9. Delirium;  10. Inability to communicate in English. Communication in languages other than English, because many of the primary assessment tools have not been validated in other languages.  11. Clinically significant sensory impairment. Auditory acuity, and visual acuity that allows for reading words, are necessary for testing global cognitive performance.  12. Recent history of cardiovascular, peripheral vascular events, or stroke.  13. High risk for suicide (e.g., active suicidal ideation and current intent or plan) in an ambulatory patient. | |  |
| iTAB-CVPilot | 1.Participants will have a clinical diagnosis of BD for at least 2 years as determined by a standardized diagnostic interview, the Mini-International Neuropsychiatric Interview (MINI) (52)  2. Have stage 1 or 2 HTN with a systolic pressure ≥130  3. Carry a diagnosis of HTN per patient self-report ≥ 6 months prior to enrollment  4. Have been prescribed at least one regularly scheduled antihypertensive medication for ≥ 3 months since diagnosis  5. Have self-reported poor adherence to at least one antihypertensive medication defined as missing 20% or more of medication within either the past week or past month (53-56) as identified by the Tablets Routine Questionnaire (TRQ)  6. Be able to participate in psychiatric interviews | 1. Unable or unwilling to participate in psychiatric interviews. This will include individuals, who may be too psychotic to participate in interviews/rating scales  2. Unable or unwilling to give written informed consent to study participation  3. Under the age of 21  4. In the interest of patient safety, individuals who are at high immediate risk for suicide will be excluded from study participation. The suicide risk assessment will be informed by standardized assessments of psychiatric symptoms and the Mini-International Neuropsychiatric Interview (MINI). In the event that a potential study participant is determined to be at high risk for suicide, that individual will not be enrolled and the study staff will immediately implement procedures for the safety of the individual. Once such individuals are deemed stable, they may be once again considered for inclusion in the research.  5. Individuals who are monolingual, non-English speaking will be excluded. Given the relatively small sample size in this study, it would not be practical to conduct sub-group analyses. Also, the study assessment tools are not available in other languages and would be impractical to develop. Based upon our BD adherence work, which drew upon a populations similar to this trial, there were no potential subjects who were excluded from the studies due to inability to speak English.  6. Illiterate participants will be excluded because reading is an essential skill required to complete self-report questionnaires administered during the study as well as to utilize the text messaging intervention.  7. Unwillingness to receive text messages | |  |
| Konya | **BSDS** (n=13)  1. Patients with bipolar disorder (type-I and type-II) having at least completed primary school and being aged between 18 and 65 years who were in remission were subsequently included in the study. 2.Remission criteria were determined as scoring less than YMRS score<5 HAMDRS-17 score<7  **QRISK** (n=16)  1.Individuals of aged between 25-84 years old  2.Individuals with bipolar disorder in remission for at least 2 months will be recruited in the outpatient bipolar mood clinic  3.Able and willing to give informed consent  4.YMRS score <6 and HAMDRS-17 score <8 | **BSDS**  1.Unable or unwilling to participate in psychiatric interviews.  2.Patients with physical or mental limitations at a level hindering them to follow the instructions or patients who met the diagnostic criteria of any type of mental interviews/rating scales  3.Unable or unwilling to give written, informed consent to study participation  4.bipolar disorder diagnosis other than bipolar type-I and type-II disorders according to DSM-IV  **QRISK**  1.Patients younger than 25 years or above the age of 84  2.Patients with current heart disease or stroke diagnoses | |  |
| M&M | 1. Over 18 years of age  2. Bipolar disorder as their Axis 1 diagnosis | See inclusion criteria. | |  |
| FNPHLag | 1.Individuals with Bipolar Disorder, aged 50 years or older  2.Clinical diagnosis of BPD based on MINI | 1.Intellectual disability or dementia  2.Unable to safely participate in clinical interview | |  |
| MOODINFLAME | BD patients:  1.18-65 years  2.Both male and female  3.Bipolar I disorder or Bipolar II disorder  4.Preferentially euthymic (IDS-C <22 and YMRS <12)  5.No other primary major psychiatric diagnosis (e.g. primary psychotic disorder, schizo-affective disorder, primary anxiety disorder)  6.No current severe alcohol or other substance use disorder, needing treatment in a specialized setting  7.No alcohol or other substance dependence in the last year  8.No current or recent (last 4 weeks) severe infectious or inflammatory disease  9.No known current uncontrolled systemic disease (e.g. LE, RA)  10.No known major uncontrolled metabolic disorder (e.g. diabetes, hyper- or hypothyroidism, Cushing disease of Addison disease)  11.No known other significant uncontrolled somatic/organic/neurological disorder which may cause/affect mood  12.No current or recent (last month) use of somatic medication which may affect mood or the immune system (e.g. corticoids, anti-inflammatory drugs, immune suppressive drugs)  13.Women: Not pregnant or recent (<6 months) delivery (will be asked; in case of doubt the woman will not be included)  14.Informed consent  Bipolar I disorder or Bipolar II disorder, who fulfill the BD-patient criteria described above except for the criterion of being preferentially euthymic and who are in a manic or depressive episode as defined by DSM IV-tr | See inclusion criteria | |  |
| PROMAN | **CBT Inclusion:**  1.18 to 55 years of age  2.Diagnosis of Bipolar Disorder type I or II based on the Structured Clinical Interview for DSM IV (SCID)  3.Able and willing to give informed consent  4.Being literate  **Creatine Inclusion:**  1.18 to 60 years of age  2.Diagnosis of Bipolar Disorder type I based on the Structured Clinical Interview for DSM IV (SCID) - current depressive episode: score > 19 on the Montgomery-Asberg Depression Rating Scale (MADRS)  3.More than two weeks of treatment with lithium (serum level> 0.8 mEq / L), valproate (serum levels> 50 mg / L) or quetiapine (300-600mg/dia dose) or drug combination; Antipsychotics, anticonvulsants, benzodiazepines, and thyroid supplementation allowed if the dose has remained stable over the past two weeks; antidepressants allowed if the dosage has remained stable for 4 weeks.  4.Able and willing to give informed consent  5.Being literate | **CBT Exclusion:**  1.IQ higher than 80  2.substance or alcohol abuse/dependence in the last 6 months  3.current suicide risk  4.organic mental disorder  5.score higher than 12 in the Montgomery Asberg Depression Scale (MADRS)  6.a score higher than 8 in the Young Mania Rating Scale (YMRS)  **Creatine Exclusion:**  1. Substance or alcohol abuse/dependence in the last 6 months  2. Current suicide risk  3. Organic mental disorder  4. Diagnosis of schizophrenia  5. Dementia  6. Delirium  7. Epilepsy  8. Mental retardation  9. Clinically unstable medical illnesses  10. Preexisting renal disease,  11. History of hypersensibility to creatine.  12.High risk for suicidal or homicidal behavior or self-mutilation  13. Women with gestational potential can only not using reliable contraception. | |  |
| TTIM | 1. Be receiving treatment for schizophrenia/schizoaffective disorder, bipolar disorder or depression (mood stabilizing, anti-psychotic or antidepressant medication);  2. Have a diagnosis of schizophrenia, schizoaffective disorder, bipolar disorder or depression; The investigators will seek confirmatory report from a health care provider that the individual has or may have SMI.  SMI diagnosis will be confirmed with the Mini-International Neuropsychiatric Interview (MINI).  3. Have Type 2 DM based upon either previous diagnosis or laboratory values;  7. Be ≥ 18 years of age;  5. Be able to communicate in English; and  6. Be able to provide written, informed consent to participation. Individuals with guardians of person may participate with guardian written consent and patient assent. | 1. Actively suicidal/homicidal;  2. Unable to be rated on study rating scales;  3. Demented;  4. Pregnant;  5. Unable to participate in groups due to uncontrolled/severe psychiatric symptoms or  6. Unable to provide informed consent.  7. Participation in another randomized clinical trial that may affect diabetes or mental health outcomes  8. Past participation in TTIM Intervention Pilot Study  9. Limited expected lifespan  10. Special physical and/or dietary needs that are not consistent with the TTIM Intervention | |  |
| UTH_Houston | **Genomics of Bipolar Disorder**  1. Gender: Both  2. Age: Adult (18-65 yrs.)  3. Ethnicity: All Ethnicities  4. Primary Language: English  5. Groups to be recruited will include: Both patients and healthy controls  *Bipolar Disorder patients:*  1. Bipolar I or II disorder diagnosed by SCID-I, according to DSM-IV-R diagnostic criteria  2. Any current mood state  3. Any comorbid psychiatric disorder, except for current substance abuse or dependence (see exclusion criteria)  *Healthy Controls:*  1. No lifetime diagnosis of a major psychiatric (mood, anxiety, psychotic, addictive) and neurological. 2. No family history of any axis I psychiatric disorder and/or hereditary neurological disorders  in first-degree relatives  *Both Bipolar Disorder patients and healthy controls:*  1. Men or women, any race/ethnicity, 18-65 years old  2. Speak English well enough to read and understand consent documents and rating scales  3. Have capacity to give informed consent disorders according to DSM-IV-R.  **In Vivo Brain Mechanisms Across the Bipolar Spectrum**  *BD patients across the 4 patient groups are:*  1. Diagnosis of BO I, II, NOS or cyclothymia based on DSM-IV criteria  2. Ages 18-65 years old   3. Not currently in an acute illness episode (HAMD < 12 and YMRS < 12)  d) Off all psychotropic medications for at least two weeks.  *The healthy controls:*  1. No lifetime or present history of any axis I psychiatric disorder, based on DSM­ IV criteria  2. Ages 18-65 years old  3. No use of any psychoactive medications in the past two weeks before enrollment in the study.  **Searching for Endophenotypes of Bipolar Disorder**  *BD sibling pairs:*  1. BD proband with diagnosis of BD I or II, based on DSM-IV criteria  2. Having a same-gender sibling not affected by BD  3. ages 18-65 years old  4. BD proband and unaffected sibling no more than 10 years apart in age  5. BD proband at any current mood state at the time of the study  6. BD proband preferably off pharmacological treatment at the time of study, but if not feasible, being on antidepressants and mood stabilizers (including anticonvulsants, typical and atypical  antipsychotics, and lithium will be allowed  7. BD proband and unaffected sibling brought up together in the same family.  *Healthy Controls:*  1. Being mentally healthy, defined as no lifetime or present history of any axis I psychiatric disorder including alcohol or substance abuse/dependence, based on DSM-IV criteria  2. Ages 18-65 years old.  **Stanley**  1. Age 18 to 65 years  2. A diagnosis of BD type I or II according to SCID-I interview;  3. Currently in a depressive or mixed episode, based on DSM-IV/ SCID-I criteria;  4. MADRAS >20 at entry in the study;  5. No CURRENT liver, kidney, heart disease or ulcers or bleeding dyscrasia;  6. No HYSTORY of kidney dysfunction or cardiac problems;  7. ON therapeutic doses of a mood stabilizing drug (lithium, anticonvulsants, any atypical antipsychotics) or combinations for at least ONE month.  8. Allowed psychiatric co-morbid conditions, such as anxiety disorders, PTSD and substance use (as long as do NOT meet abuse or dependence criteria according to the SCID-I in the past 2 months). | **Genomics of Bipolar Disorder**  *Psychiatric Healthy Controls:*  1. History of any axis I psychiatric disorder in first-degree relatives  *Bipolar Disorder Patients:*  1. Patients that currently meet for substance abuse or dependence, defined as meeting DSM-IV-R criteria for substance abuse or dependence within the last 6 months will be excluded only for the MRI and EEG studies. *Medical Healthy Controls or Bipolar Disorder patients:*  1. Current major medical problems that affect brain anatomy, neurochemistry, or function, e.g., diabetes, hypertension, liver insufficiency, kidney insufficiency, cardiovascular problems, systemic infections, cancer, hypothyroidism, auto-immune diseases, and any brain disorder (seizure disorder, stroke, dementia, degenerative neurologic diseases), per investigators judgement.  2. History of any brain diseases, including seizures, stroke, meningitis, encephalitis, dementia, degenerative brain diseases, and head injury with loss of consciousness for any period of time.  3. Family history of hereditary neurologic disorder  4. Pregnancy 3)  *General Healthy Controls and Bipolar Disorder patients:* 1. Unable to give informed consent for any reason  *Neuroimaging-specific Healthy Controls and Bipolar Disorder patients:*  1.Floating metallic objects in the body  *Bipolar Disorder Patients:*  1. Current substance abuse or dependence, defined as meeting DSM-IV-R criteria for substance  abuse or dependence within the last 6 months  **In Vivo Brain Mechanisms Across the Bipolar Spectrum**  *BD patients across the 4 patient groups:*  1. Presence of any axis I psychiatric comorbid disorder, except from anxiety disorders, binge eating disorder and substance disorders in remission for at least six months  2. Current major medical problems  3. History of neurologic disorders, including head injury with loss of consciousness  4. Family history of hereditary neurologic disorders; and 5. Floating metallic objects in the body (exclusion for MR studies).  *The healthy controls:*  1. history of any axis I psychiatric disorder in first-degree relatives;  2. current major medical problems  3. history of neurologic disorders, including head injury with loss of consciousness  4. family history of hereditary neurologic disorders  5. Floating metallic objects in the body (exclusion for MR studies).  **Searching for Endophenotypes of Bipolar Disorder**  *BD sibling pairs:*  1. Diagnosis of Bipolar Disorder, Schizoaffective Disorder or Schizophrenia is not allowed. Alcohol and substance abuse/ dependence (if in remission in the past 6 months)  and anxiety disorders are allowed  2. Being on a regular dose of benzodiazepines within two weeks of study  participation  3. Pregnancy  4. Ineligibility or inability of one of the members of the sibling pair to participate in the study.  *Healthy Controls:*  1. History of any axis I psychiatric disorder in first-degree relatives  2. Having taken a prescribed psychotropic medication at any point in their lives.  *Both BD sibling pairs and healthy controls:*  1. Current major medical problems that affect brain anatomy, neurochemistry, or function, e.g., diabetes, hypertension, liver insufficiency, kidney insufficiency, cardiovascular problems, systemic infections, cancer, hypothyroidism, auto-immune diseases, and any brain disorder (seizure disorder, stroke, dementia, degenerative neurologic diseases);  2. History of any brain diseases, including seizures, stroke, meningitis, encephalitis, dementia, degenerative brain diseases, and head injury with loss of consciousness for any period of time  3.Family history of hereditary neurologic disorder  4. Floating metallic objects in the body (exclusion for MR studies)  5. Pregnancy  **Stanley**  1. CANNOT be on any :  Anti-inflammatory: NSAIDs:  • Aspirin (bufferin, bayer aspirin, ecotrin),  • diflunisal (dolobid, diflunisal),  • Salsalate (amigesic, salflex),  • Ibuprofen (motrin, advil),  • Naproxen (naprosyn,aleve, midol extended relief),  • Fenoprofen (nalfon),  • Ketoprofen (actron),  • dexketoprofen(ketron D),  • Flurbiprofen (ansaid),  • Oxaprozin (daypro),  • Loxoprofen (loxfen, loxonin),  • Indomethacin (indocin, indocin SR),  • Silindac (clinoril), Etodolac (lodine),  • Ketorolac (toradol), diclofenac (voltaren, cataflan),  • Nabumetone (Relafen)  • Piroxicam (feldene),  • Meloxicam (mobic),  • Tenoxicam (mobiflex),  • Lornixicam (xefo),  • mefenamic acid (ponstel),  • meclofenamic acid (meclofenamate sodium),  • celecoxib (celebrex)  Anticoagulants:  • Coumadin (Warfarin), Heparin  • Anti-oxidant agents  • Fish oil  • NAC ( N-acetyl cysteine)  2. Pregnancy  3. CANNOT change the dose of the psychotropic medications during the trial | |  |
| UTH_SanAntonio | **A Family Study of Brain Abnormalities in Bipolar Disorder**  *Bipolar subjects:*  1. Diagnosis of Bipolar Disorder type I according to DSM-IV  2. age 18 or over  3. No alcohol or substance abuse/dependence within 6 months of study participation  4. No current major medical problems.  *Unaffected relatives of bipolar subjects:*  1. No diagnosis of bipolar disorder type I according to DSM-IV  2. Age 18 or over  3. No alcohol or substance abuse/dependence within 6 months of study participation  4. No current major medical problems.  *Healthy individuals without a family history of psychiatric disorders:* 1. Age 18 or over  2. No current major medical problems  3. No current psychiatric or neurologic disorder  4. No history of psychiatric disorders on a first-degree relative. Subject population will include both genders and minority groups representative of the local community.  **Brain Myelin Integrity in Bipolar Disorder An In Vivo Magnetic Resonance Imaging (MRI) Study**  *Bipolar subjects:*  1. Age 18-75  2. DSM-IV diagnosis of bipolar disorder type I  3. No substance abuse within the past 6 months  4. No current medical problems  5. Not receiving psychotropic medications for at least two weeks at the time of participation in the study  6. No comorbid psychiatric disorders, except for Anxiety Disorders.  *Healthy individuals:*  1. Age 18-75  2. No current medical problems  3. No history of current psychiatric or neurologic disorder  4. No history of mental illness in a first-degree relative.  Subject population will include both genders and minority groups representative of the local community.  **Celecoxib**  1. Experiencing a major depressive or mixed episode  2. A HAMD score ≥18  3. previously been on therapeutic and regular doses of a mood stabilizer or atypical antipsychotic for at least I month, and did not adequately respond toil. Subjects were allowed to remain on benzodiazepines, antidepressants, and stimulants.  **In Vivo Brain Mechanisms Across the Bipolar Spectrum**  *BD patients across the 4 patient groups:*  1. Diagnosis of BO I, II, NOS or cyclothymia based on DSM-IV criteria  2. Ages 18-65 years old  3. Not currently in an acute illness episode (HAMD < 12 and YMRS < 12)  4. Off all psychotropic medications for at least two weeks.  *The healthy controls will be matched to the patients for age, gender, race and years of education:*  1. No lifetime or present history of any axis I psychiatric disorder, based on DSM­ IV criteria  2. Ages 18-65 years old  3. No use of any psychoactive medications in the past two weeks before enrollment in the study.  **In Vivo Brain Mechanisms in Comorbid Bipolar Disorder and Alcoholism**  *Patients:*  1. Ages 18-75  2. DSM-IV diagnosis  Bipolar Type I  3. Alcohol abuse or dependence, according to DSM-IV (for the 30 patients with comorbid alcoholism)  4. No current medical illnesses or neurological disorders  5. No other Axis I DSM-IV psychiatric disorders.  *Healthy controls:*  1. Ages 18-75  2. No prior or current psychiatric or neurological illnesses  3. No first-degree relatives with a psychiatric disorder.  **In Vivo Brain Mechanisms Underlying Remission in Bipolar Depression**  Specific inclusion and exclusion criteria are:  *Patients:*  1. Ages 18-75  2. DSM-IV diagnosis of Bipolar Type I  3. Alcohol abuse or dependence, according to DSM-IV (for the 30 patients with comorbid alcoholism)  4. No current medical illnesses or neurological disorders  5. No other Axis I DSM-IV psychiatric disorders.  *Healthy controls:*  1. Ages 18-75  2. No prior or current psychiatric or neurological illnesses  3. No first-degree relatives with a psychiatric disorder.  **In Vivo Studies of the phosphoinositol (PI) Pathway in Bipolar Disorder**  *Bipolar subjects - depressed:*  1. Age 18-65  2. Free of any psychotropic drugs for at least two weeks  3. No psychiatric comorbidities  4. No current medical problems  5. Depression score of at least 18 in the Hamilton Depression Rating Scale (HDRS-17) items.  *Bipolar subjects - manic:*  1. Age 18-65  2. Free of any psychotropic drugs for at least two weeks  3. No psychiatric comorbidities  4. No current medical problems  5. Manic scores of at least 14 in the Young Mania Rating Scale (YMRS).  *Bipolar subjects - euthymic:*  1. age 18-65  2. free of any psychotropic drugs for at least two weeks  3. no psychiatric comorbidities  4. no current medical problems  5. Asymptomatic for over a month - depression score < 4 in the HORS, and < 4 in the YMRS at time of entry in the study.  *Bipolar subjects - euthymic, lithium-treated:*  1. age 18-65  2. free of other psychotropic drugs other than lithium for at least two weeks  3. no psychiatric comorbidities  4. no current medical problems  5. asymptomatic for over a month- depression score < 4 in the HDRS, and < 4 in the YMRS at time of entry in the study  6. On lithium treatment at therapeutic serum levels (0.6-1.2 mEq/L) for over a month.  *Normal comparison subjects:*  1. age 18-65 and  2. No history of or current psychiatric or neurologic disorder. Healthy subjects, upon signing the consent form, will be submitted to a detailed interview using the Structured Clinical Interview for Diagnosis Non­ Patient version (SCIO-NP) to rule out DSM-IV Axis I disorders.  **Neuroanatomy in Bipolar Disorder**  1. Age 18-65 years old  2. Off all psychotropic medications for at least two weeks  3. In a depressed episode of the illness (HAMD 21 items ≥ 15)  4. No significant manic symptoms (YMRS < 12) at entry in the study 5. No comorbid alcohol or substance abuse or dependence in the last six months.  *healthy control subjects, who will be matched to BD patients for age and gender:*  1. Age 18-65 years old  2. No history of lifetime psychiatric or neurologic disorder  3. No use of any prescribed psychiatric medications in their lifetimes. Healthy subjects will be screened initially via a preliminary telephone interview conducted by the study coordinator, and if they appear to qualify, they will come in for a detailed interview using the SCID-IV (patient version) to rule out DSM-IV Axis I disorders.  **A Family Study of Brain Abnormalities in Unipolar Disorder**  *Unipolar subjects:*  1. Age 18 or over  2. No current major medical problems  3. Diagnosis of recurrent unipolar disorder according to Diagnostic and Statistical Manual of Mental Disorders (DSM-IV), as determined by Structured Clinical Interview for Diagnosis (SCID-IV) interview; 4. No alcohol or substance abuse/dependence within 6 months of study participation; e) have an eligible sibling who wants to participate in this study.  *Non-affected relatives of unipolar subjects:*  1. No diagnosis of Unipolar Disorder type I according to DSM-IV  2. Age 18 or over  3. No alcohol or substance abuse/dependence within 6 months of study participation  4. No current major medical problems.  Healthy individuals without a family history of psychiatric disorders:  1. Age 18 or over  2. No current major medical problems  3. No history of current psychiatric or neurologic disorder  4. No history of mental illness on a first-degree relative.  **Brain imaging Investigations of Unipolar Depressed Patients**  Unipolar subjects:  1. age 18-65  2. free of any psychotropic drugs for at least two weeks  3. no psychiatric comorbidities, with the exception of anxiety disorders  4. no current medical problems  5. Depression score of at least 18 in the Hamilton Depression Rating Scale (HDRS-21) items for the depressed group, and no more than 4 for the remitted group at time of entry in the study. Remitted patients should not have met criteria for a major depressive episode within the past 6 months at the time of entry in the study.  *Normal comparison subjects:*  1. age 18-65  2. No history of or current psychiatric or neurologic disorder. Healthy subjects, upon signing the consent form, will be submitted to a detailed interview using the Structured Clinical Interview for Diagnosis-Non­ Patient version (SCID-NP) to rule out DSM-IV Axis I disorders. | **A Family Study of Brain Abnormalities in Bipolar Disorder**  1. Pregnancy,  2. Neurologic disorders, including head injury with loss of consciousness, family history of hereditary neurologic disorder, or floating metallic objects in the body (exclusion for Magnetic Resonance [MR] studies).  3. Subjects who test positive for illegal substances will be excluded, because current substance use could be related to specific brain changes.  **Brain Myelin Integrity in Bipolar Disorder An In Vivo Magnetic Resonance Imaging (MRI) Study**  Patients and healthy controls:  1. Pregnancy,  2. Neurologic disorders, including head injury with loss of consciousness,  3. Family history of hereditary neurologic disorder,  4. Floating metallic objects in the body (exclusion for MR studies).  **Celecoxib**  1. Current liver, kidney, heart disease. or chronic pain,  2. Prior history of kidney dysfunction or cardiac problems,  3. Prior history of sensitivity to sulfonamides, current use of a non-steroidal anti-inflammatory agent,  4. Prior history of gastric ulcers, presence of risk factors for or prior history of cardiovascular or thromboembolic diseases (such as hypertension, diabetes, hypercholesterolemia, stroke, coronary artery disease)  5. Pregnancy.  **In Vivo Brain Mechanisms Across the Bipolar Spectrum**  *BD patients across the 4 patient groups:*  1. Presence of any axis I psychiatric comorbid disorder, except from anxiety disorders, binge eating disorder and substance disorders in remission for at least six months  2. Current major medical problems  3. History of neurologic disorders, including head injury with loss of consciousness  4. Family history of hereditary neurologic disorders; and  5. Floating metallic objects in the body (exclusion for MR studies).  *The healthy controls:*  1. History of any axis I psychiatric disorder in first-degree relatives;  2. Current major medical problems  3. History of neurologic disorders, including head injury with loss of consciousness  4. Family history of hereditary neurologic disorders  5. Floating metallic objects in the body (exclusion for MR studies).  **In Vivo Brain Mechanisms in Comorbid Bipolar Disorder and Alcoholism**  1. Neurologic disorders, including head injury with loss of consciousness,  2. Family history of hereditary neurologic disorder, or floating metallic objects in the body (exclusion for MR studies).  3. Patients' alcohol abuse or dependence should be active within the month preceding the MRI/MRS scan, and they need to have met DSM-IV criteria for alcohol dependence or abuse for at least one year.  4. Patients could be in any mood state at the point they are enrolled (manic, mixed, euthymic, or depressed),  5. Either untreated or currently in treatment with psychotropic medications.  **In Vivo Studies of the phosphoinositol (PI) Pathway in Bipolar Disorder**  *The unmedicated patients:*  1. Be drug-free for at least two weeks.  2. Not have any comorbid psychiatric disorder, no current medical problems, and no alcohol or substance abuse within the past 6 months.  3. Pregnancy (Pregnancy will be ruled out by history, and if needed, by doing a serum pregnancy test.)  4. Neurologic disorders, including head injury with loss of consciousness  family history of hereditary neurologic disorder, or floating metallic objects in the body (exclusion for magnetic resonance [MR] studies).  *Normal comparison subjects:*  1. history of any Axis I psychiatric disorder in first-degree relatives  2. treatment with any psychoactive medication within six months of baseline assessment  3. current medical problems. Healthy control subjects will NOT receive lithium in this study.  **Neuroanatomy in Bipolar Disorder**  1. Alcohol or other substance abuse or dependence if not in remission for the last 6 months)  2. Use of any psychoactive medication within two weeks prior to entry in the study  3. Current major medical problems  4. Previous history of neurologic disorders, including head injury with loss of consciousness for any period of time  5. Pregnancy  6. Family history of hereditary neurologic disorder  7. Floating metallic objects in the body (exclusion for MR studies).  Healthy control:  1. History of any Axis I psychiatric disorder in first-degree relatives  2. Use of any prescribed psychiatric medication in their lifetimes  3. Current major medical problems  4. Previous history of neurologic disorders, including head injury with loss of consciousness for any period of time  5. Pregnancy  6. Family history of hereditary neurologic disorder  7. Floating metallic objects in the body (exclusion for MR studies).  **A Family Study of Brain Abnormalities in Unipolar Disorder**  1. Pregnancy,  2. Neurologic disorders, including head injury with loss of consciousness,  3. Family history of hereditary neurologic disorder, or floating metallic objects in the body (exclusion for magnetic resonance [MR] studies).  4. Subjects who test positive for illegal substances will be excluded, because current substance use could be related to specific brain changes.  **Brain imaging Investigations of Unipolar Depressed Patients**  *Normal comparison subjects:*  1. History of any Axis I psychiatric disorder in first-degree relatives  2. Treatment with any psychoactive medication within six months of baseline assessment  3. Current medical problems. | |  |
| UTH_UNC | **A Family Study of Brain Abnormalities in Bipolar Disorder**  *Bipolar subjects:*  1. Diagnosis of Bipolar Disorder type I according to DSM-IV  2. age 18 or over  3. No alcohol or substance abuse/dependence within 6 months of study participation  4. No current major medical problems.  Unaffected relatives of bipolar subjects:  1. No diagnosis of bipolar disorder type I according to DSM-IV  2. Age 18 or over  3. No alcohol or substance abuse/dependence within 6 months of study participation  4. No current major medical problems.  *Healthy individuals without a family history of psychiatric disorders:* 1. Age 18 or over  2. No current major medical problems  3. No current psychiatric or neurologic disorder  4. No history of psychiatric disorders on a first-degree relative. Subject population will include both genders and minority groups representative of the local community.  **In Vivo Brain Mechanisms Across the Bipolar Spectrum**  *BD patients across the 4 patient groups:*  1. Diagnosis of BO I, II, NOS or cyclothymia based on DSM-IV criteria  2. Ages 18-65 years old  3. Not currently in an acute illness episode (HAMD < 12 and YMRS < 12)  4. Off all psychotropic medications for at least two weeks.  *The healthy controls will be matched to the patients for age, gender, race and years of education:*  1. No lifetime or present history of any axis I psychiatric disorder, based on DSM­ IV criteria  2. Ages 18-65 years old  3. No use of any psychoactive medications in the past two weeks before enrollment in the study.  **Neuroanatomy in Bipolar Disorder**  1. Age 18-65 years old  2. Off all psychotropic medications for at least two weeks  3. In a depressed episode of the illness (HAMD 21 items ≥ 15)  4. No significant manic symptoms (YMRS < 12) at entry in the study 5. No comorbid alcohol or substance abuse or dependence in the last six months.  *healthy control subjects, who will be matched to BD patients for age and gender:*  1. Age 18-65 years old  2. No history of lifetime psychiatric or neurologic disorder  3. No use of any prescribed psychiatric medications in their lifetimes. Healthy subjects will be screened initially via a preliminary telephone interview conducted by the study coordinator, and if they appear to qualify, they will come in for a detailed interview using the SCID-IV (patient version) to rule out DSM-IV Axis I disorders. | **A Family Study of Brain Abnormalities in Bipolar Disorder**  1. Pregnancy,  2. Neurologic disorders, including head injury with loss of consciousness, family history of hereditary neurologic disorder, or floating metallic objects in the body (exclusion for Magnetic Resonance [MR] studies).  3. Subjects who test positive for illegal substances will be excluded, because current substance use could be related to specific brain changes.  **In Vivo Brain Mechanisms Across the Bipolar Spectrum**  *BD patients across the 4 patient groups:*  1. Presence of any axis I psychiatric comorbid disorder, except from anxiety disorders, binge eating disorder and substance disorders in remission for at least six months  2. Current major medical problems  3. History of neurologic disorders, including head injury with loss of consciousness  4. Family history of hereditary neurologic disorders; and  5. Floating metallic objects in the body (exclusion for MR studies).  *The healthy controls:*  1. History of any axis I psychiatric disorder in first-degree relatives;  2. Current major medical problems  3. History of neurologic disorders, including head injury with loss of consciousness  4. Family history of hereditary neurologic disorders  5. Floating metallic objects in the body (exclusion for MR studies).  **Neuroanatomy in Bipolar Disorder**  1. Alcohol or other substance abuse or dependence if not in remission for the last 6 months)  2. Use of any psychoactive medication within two weeks prior to entry in the study  3. Current major medical problems  4. Previous history of neurologic disorders, including head injury with loss of consciousness for any period of time  5. Pregnancy  6. Family history of hereditary neurologic disorder  7. Floating metallic objects in the body (exclusion for MR studies).  Healthy control:  1. History of any Axis I psychiatric disorder in first-degree relatives  2. Use of any prescribed psychiatric medication in their lifetimes  3. Current major medical problems  4. Previous history of neurologic disorders, including head injury with loss of consciousness for any period of time  5. Pregnancy  6. Family history of hereditary neurologic disorder  7. Floating metallic objects in the body (exclusion for MR studies). | |  |
| VA_BAI | 1. Ages 30-79 years old.  *Patients:*  2. DSM-IV diagnosis of BD and onset of first mood episode between ages 13 to 35 years. | *Patients:*  1. Current depressive or manic episode as determined by DSM-IV criteria or significant residual mood or psychotic symptoms, change of medication or dose in past 6 weeks, other co-morbid Axis I disorder (anxiety disorder allowed if no symptoms or treatment within 1 year).  2. Current or recent (past 6 months for abuse, past 12 months for dependence) diagnosis of substance abuse or dependence,  3. History of head injury with loss of consciousness for > 30 minutes, left handedness, history of neurological disorder (e.g., seizure disorder, Parkinson’s or Alzheimer’s disease, stroke).  4. History of diabetes, uncontrolled hypertension  5. Contraindications for MRI scanning (e.g., metal in the body, weight over 300 lbs, claustrophobia, difficulty lying still, pregnancy).  6. Native language other than English.  7. Conservatorized.  8. Children under the age of 18 will be excluded due to the aims of the study to examine adult age-related changes.  *Healthy individuals:*  1. Current Axis I disorder as determined by the Mini-International Neuropsychiatric Interview (MINI 4.0), first-degree relatives with bipolar disorder, unipolar depression, or schizophrenia.  2. All the non-psychiatric exclusion criteria listed above. |  |  |
| Vienna (SFU) (Neumnkirchen Cohort) | 1. A diagnosis of bipolar I and bipolar II (MINI, and 2 trained Psychiatrists clinical diagnosis coded ICD-10).  2. Age>18 years old  3. Provided informed consent for database and contact for follow up.  4. Unselected consecutive hospitalized patients. | The presence of neurologic diseases in participants and intellectual disability (IQ < 70) were the only excluding criteria. | |  |
| VOCS_BD | 1.Adults (age 18 -80,inclusive)  2.DSM5 Diagnosis of Type I or type II Bipolar Disorder, confirmed with the Structured Clinical Interview for DSM (SCID).  3.Outpatients attending the Alzira Mental Health Unit, affiliated with La Ribera University Hospital, Valencia, Spain.  4.Euthymia, defined as HRSD 17-item total score <8 and YMRS total score <6.5.Able to understand study procedures and willing to provide informed consent. | 1.Age older than 80 years  2.Intellectual disability  3.Cognitive impairment due to dementia (DSM5 criteria of Major Neurocognitive Disorder)  4.Electro convulsive therapy (ECT) in the previous 12 months.  5.History of head trauma.  6.Comorbid DSM-5-defined severe personality disorder or substance use disorder (except nicotine)  7.Having any sensory impairments that could interfere with test performance. | |  |
